# Prospective of mycorrhiza and *Beauvaria bassiana* silica nanoparticles on *Gossypium hirsutum* L. plants as biocontrol agent against cotton leafworm, *Spodoptera littoralis*

**DOI:** 10.1186/s12870-022-03763-x

**Published:** 2022-08-20

**Authors:** Rabab A. Metwally, Hala Sh. Azab, Hatem M. Al-Shannaf, Gamal H. Rabie

**Affiliations:** 1 Botany and Microbiology Department, Faculty of Science, Zagazig University, Zagazig, Egypt; 2 Plant Protection Research Institute, Agriculture Research Center, Giza, Egypt

**Keywords:** Antioxidant enzymes, Arbuscular mycorrhizal fungi, Biocontrol, *Gossypium hirsutum*, *Spodoptera littoralis*, Plant-microbe-insect interaction, Proline

## Abstract

**Background:**

Plant-herbivorous insects are a severe danger to the world’s agricultural production of various crops. Insecticides used indiscriminately resulted in habitat destruction due to their high toxicity, as well as disease resistance. In this respect, the development of a sustainable approach to supreme crop production with the least damage is a crucially prerequisite. As a result, the current study was carried out to understand the potential effect of arbuscular mycorrhizal (AM) fungi along with *Beauvaria bassiana* silica nanoparticles (Si NPs) as a new approach to increase cotton (*Gossypium hirsutum* L. Merr.) defense against an insect herbivore, *Spodoptera littoralis*. AM and non-AM cotton plants were infested with *S. littoralis* and then sprayed with a biopesticide [*B. bassiana* Si NPs] or a chemical insecticide (Chlorpyrifos).

**Results:**

The gas chromatography-mass spectrometry (GC–MS) analysis of *B. bassiana* Si NPs fungal extract showed that the major constituents identified were Oleyl alcohol, trifluoroacetate, 11-Dodecen-1-AL and 13-Octadecenal, (Z)-(CAS). Besides, results revealed a highly significant decrease in growth parameters in *S. littoralis* infested plants, however, with AM fungal inoculation a substantial improvement in growth traits and biochemical parameters such as protein and carbohydrates contents was observed. In addition, stimulation in proline and antioxidant enzymes activity and a decrease in malondialdehyde content were observed after AM inoculation.

**Conclusion:**

AM fungi mitigate the harmful effects of herbivorous insects by strengthening the cotton plant’s health via enhancing both morphological and biochemical traits that can partially or completely replace the application of chemical insecticides.

## Highlights

• Interactions between arbuscular mycorrhizal (AM) fungi and *Beauvaria bassiana* metabolites applied with silica nanoparticles (Si NPs) in biocontrol of herbivorous insects of cotton plants are first studied.

• Oleyl alcohol, trifluoroacetate, 11-Dodecen-1-AL and 13-Octadecenal, (Z)-(CAS) were the major compounds in ethyl acetate extract detected by Gas chromatography-mass spectrometry (GC–MS) analysis.

• Inoculation of cotton plants with AM fungi promotes growth traits of cotton plants and enhances their tolerance to *Spodoptera littoralis* insect.

*• S. littoralis* caused a reduction in AM colonization rates; besides with Chlorpyrifos application, a higher decrease was reported.

• The cotton leafworm causes stimulation of lipid peroxidation in cotton leaves, and with AM fungi and/or *B. bassiana* Si NPs this increase is reduced greatly.

• AM fungi increased total soluble protein, carbohydrates, proline content and antioxidant enzymes in cotton plant leaves as a result of *S. littoralis* infestation.

## Background

Cotton (*Gossypium hirsutum* L.) is an important natural fiber and economic crop that provides substantial benefits to humans [1]. In the last decades, the production of cotton suffered from an increased incidence of the Egyptian cotton leafworm, *Spodoptera littoralis*, which is one of the most destructive pests as well does serious damage to many important agricultural crops in Egypt such as groundnut, soybean, tomato, sweet potato, and tobacco [2, 3]. The development of effective control methods against *S. littoralis* is urgently needed. One of these effective methods is the application of chemical pesticides. These pesticides are the most common tool used to control pests and diseases. The pesticide manufacturing companies endorsed pesticides at a definite dose, however, pesticides dealers often propose overdosing to the farmers; these higher doses can probably harm the host crops and their associated soil beneficial microbes in agroecosystems [4, 5]. Also, there are several reports of the resistance development in *Spodoptera* sp. against a wide range of insecticides, resulting in many sporadic outbreaks of the pests which have led to the failure of crops [3, 6]. The increasing demands for the reduction of chemical inputs in agriculture and increased resistance to insecticides have given a considerable stimulus to the production of alternative forms of insect-pest control.

Biological control is another attractive alternative method to chemical pesticides due to its non-toxicity for humans and other organisms and being less environmentally harmful [7, 8], since they neither leave toxic chemical residues in the environment nor induce resistance in their insect hosts [9, 10]. Among them, entomopathogenic fungi, *Beauvaria bassiana* with a broad host range is known as an effective organism to control medical and agricultural pests [11], it is also recommended to be used as an alternative to biofertilizers in agriculture [12]. The mode of action of entomopathogenic fungi includes the production of a large array of biologically active metabolites [7, 8] such as toxic proteins, enzymes, and bioactive secondary metabolites to overawe the insect immune system and modify the host performance [13]. These secondary metabolites are alkaloids (tennelin, bassianin, pyridovericin), non-peptide pigment (oosporein), non-ribosomally synthesized cyclodepsipeptides (beauvericins and allobeauvericins, bassianolides) and cyclopeptides (beauveriolides), and other metabolites convoluted in pathogenesis and virulence that have possible or recognized industrial and agricultural uses [9, 14].

The traditional method of controlling arthropod pests with *B. bassiana* is to apply the fungal propagules directly to the plant where the pests generally develop. The success of the direct-product application is contingent on the fungus’ subsequent development and reproduction in the arthropod pest, resulting in its death. High temperatures, rainfall, and humidity, despite a variety of abiotic challenges such as low and high incidence of sunshine, function as unwanted effectors on the action of entomopathogenic fungi [15–17]. Recent studies have identified nanotechnology as an emerging science that has the potential to drastically transform the food and agricultural industries through the use of nanoparticles (NPs) for disease and insect management [3, 18]. Banu and Balasubramanian [19, 20] and Amerasan et al. [21] found that Ag NPs made from several entomopathogenic fungi (*Metarhizium anisopliae*, *B. bassiana*, and *Isaria fumosorosea*) are powerful mosquito bio-pesticides. Wang et al. [22] demonstrated the toxicity of *I. fumosorosea*-derived Fe^0^ NPs against *Bemisia tabaci*, sweet potato whitefly. Furthermore, Xu et al. [3] described the utilization of *B. brongniartii* in the synthesis of Fe^0^ NPs for the treatment of *S. litura*.

Additionally, the symbiosis of arbuscular mycorrhizal (AM) fungi with the plant roots can provide several benefits to the host such as facilitating the nutrient uptake from the soil [23–26], augmenting its growth and helping the plants better cope with abiotic and biotic stress and increase its resistance [27]. Another feature of this pervasive symbiosis implies that during the early developmental stages of the AM symbiosis, plant defense responses are modulated to facilitate AM fungal roots colonization, which then leads to activation of plant immune responses against aboveground and belowground insect herbivores both at the local level and throughout the plant [24, 28]. Also, the bio-control activity of AM fungi has been studied against various plant diseases [29–31]. AM fungi can alter plant-insect herbivorous interactions through multiple mechanisms, causing changes in plant nutrient availability, defensive strategies, and stress tolerance [24].

Consequently, the attacking of plants by herbivorous pests may rapid various types of physiological responses and oxidative damage in plants [32]; enhancing the accumulation of free radicals and reactive oxygen species (ROS) thus, causing oxidative damage [33]. Among the compounds involved in plant defense and ROS detoxification studied are antioxidants [32, 34]. Some evidence from tomatoes, coffee and other plants showed that AM colonization may change plant resistance by altering plant defense against insect herbivores and root-knot nematodes [35, 36].

Therefore, various research have been conducted to overcome the adverse effects of chemical residues on human health, such as the current study, which focuses on adopting eco-friendly alternatives such as AM fungus and *B. bassiana* Si NPs as biocontrol against *S. littoralis* on cotton plant. Nevertheless, reports dealing with their combined biocontrol activity against insect infection are limited. As a result, the study presented herein is accompanied by two hypotheses; the first was that AM fungal application enhances the growth and acts as a stimulator to increase the health efficiency of cotton plants against *S. littoralis*. The second was that *B. bassiana* Si NPs along with AM fungi increased the effectiveness of cotton plants as a biocontrol against this dangerous Egyptian pest to lessen the harmful effects of chemical pesticides.

## Results

### GC–MS analysis

**Fig. 1:**
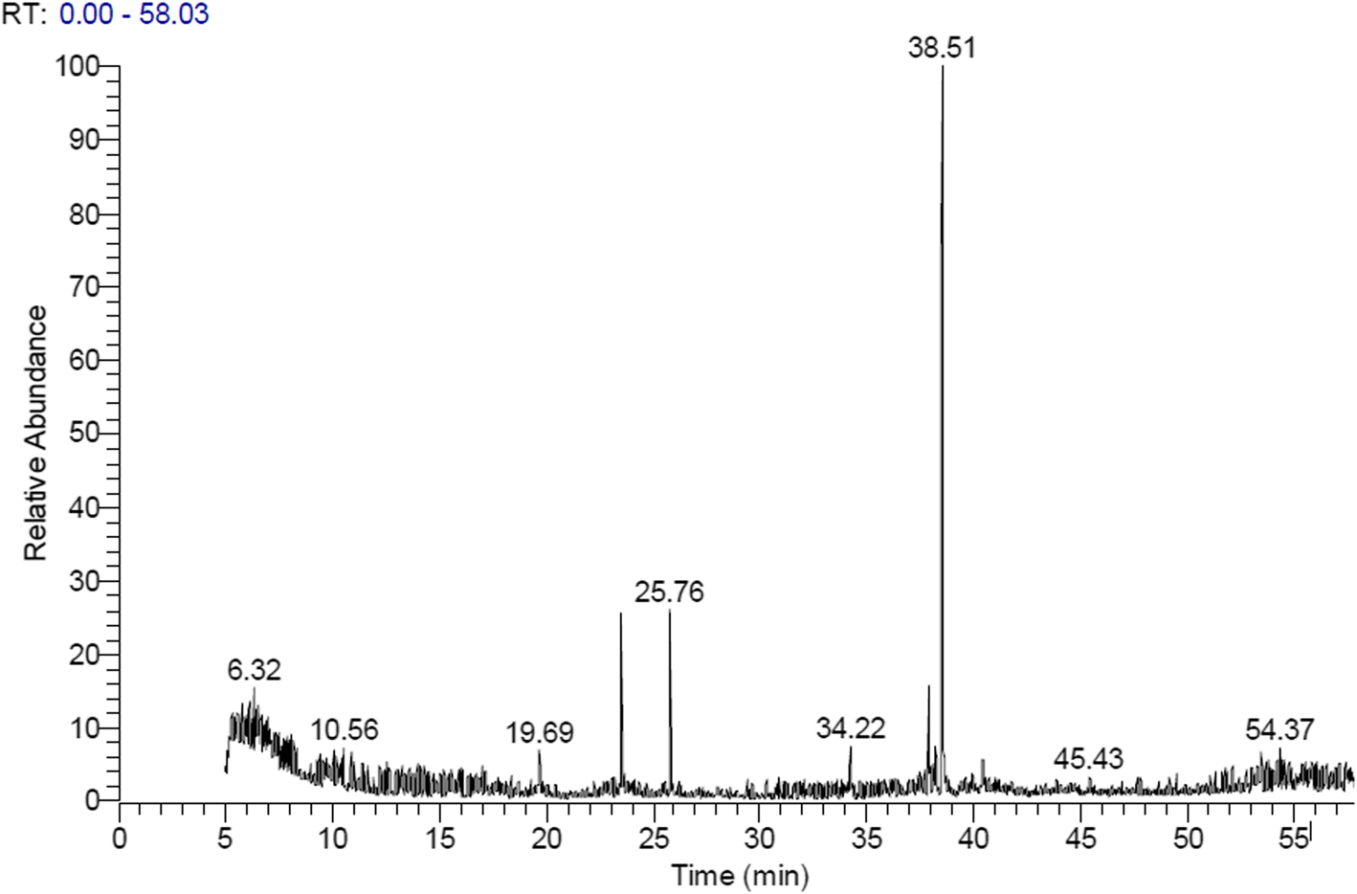
GC-MS chromatogram of bioactive compounds in ethyl acetate extract of *B. bassiana* Si NPs

Table 1 and Figure 1 represented the identified compounds from ethyl acetate extract of *B. bassiana* Si NPs. The analysis of this extract led to identification of 20 different compounds. The main constituents detected were Oleyl alcohol,trifluoroacetate (27.76%), 13-Octadecenal, (Z)-(CAS) (5.2%), 2-Docecen-1-AL (6.21%), 11-Dodecen-1-AL (2.54%), and 9-Octadecenoic acid, methyl ester- (CAS) (2%). Russuphelol (1.99%) and 2,2-Bis [4-[[4-chloro-6-(3-ethynylphenoxy) 1,3,5-triazin-2-yl] oxy] phenyl] propane (1.22%) were also detected.

**Table 1:** Bioactive compounds identified from ethyl acetate extract of *B. bassiana* Si NPs through GC-MS analysis

No.	Name of the compound	Molecular Formula	MW	Area%	RT
**1**	1,1-Dibromo −6- (triisopropylsilyl)-3,4-bis [(triisopropylsily) ethynyl] hexa-1, 3-diene-5-yne	C_37_H_64_Br_2_Si_3_	750	1.78	5.21
**2**	2-Methoxycarbonylvinyl-4-(2-bromoethyl) deutero porphyrin dimethyl ester	C_38_H_41_BrN_4_O_6_	728	1.56	5.28
**3**	1,3,6,8- tetrabromo-9-(4′-iodophenyl) carbazole	C_18_H_8_Br_4_IN	681	1.86	6.42
**4**	Russuphelol	C_26_H_16_C_l6_O_8_	666	1.99	6.54
**5**	11-Dodecen-1-AL	C_12_H_22_O	182	2.54	19.68
**6**	2-Docecen-1-AL	C_16_H_30_	222	6.21	25.77
**7**	9-Octadecenoic acid, methyl ester- (CAS)	C_19_H_36_O_2_	296	2.00	34.23
**8**	13-Octadecenal, (Z)-(CAS)	C_18_H_34_O	266	5.20	37.89
**9**	2H-Pyran, tetrahydro-2-(12- pentadecynyloxy)- (CAS)	C_20_H_36_O_2_	308	1.63	38.22
**10**	Oleyl alcohol,trifluoroacetate	C_20_H_35_F_3_O_2_	364	27.76	38.51
**11**	Chlorocarbonyl t-butoxy sulfide	C_5_H_9_C_l_O_2_S	168	1.36	40.38
**12**	Glyceryl tridocasahexonate	C_69_H_98_O	942	1.39	54.20
**13**	Delsoline	C_25_H_41_NO_7_	467	1.56	54.48
**14**	5-Chloro-2-(5,6-dinitro-1H-benzoimidazol-2-ylmethylsulfanyl) -4,6- dimethyl-nicotinonitrilel	C_16_H_11_C_l_N_6_O_4_S	418	1.28	53.43
**15**	(2-Formamido-3-nitro-5, 10,15,20 tetraphenylporphyrinato) copper (II)	C_45_H_28_CuN_6_O_3_	763	1.26	40.48
**16**	2,7,12,17-Tetraethyl-3,5: 8,10:13,15:18,20-tetrakis (2,2-dimethylpropano) porphyrin	C_48_H_62_N_4_	694	1.34	11.02
**17**	2,2-Bis [4-[[4-chloro-6-(3-ethynylphenoxy) 1,3,5-triazin-2-yl] oxy] phenyl] propane	C_37_H_24_C_l2_N_6_O_4_	686	1.22	13.24
**18**	(5,10,15,20- tetraphenyl [2-(2) H1] prophyrinato) zinx (II)	C_44_H_27_DN_4_Ni	670	1.18	14.14
**19**	1,2-15,16- Diepoxyhexadecane	C_16_H_30_O_2_	254	1.16	38.11
**20**	Pentamethyl pentaphenyl cyclo-pentasiloxane	C_35_H_40_O_5_Si_5_	680	1.10	7.52

*RT Retention time, *MW* Molecular weight

Data in Table 2 showed a highly significant effect of TLC extracted fractions of *B. bassiana* Si NPs on larval mortality percentage as compared to the control. The highest mean of larval mortality percentages was 39.84% recorded at fraction No. 3 of *B. bassiana* Si NPs; while the lowest larval mortality (10.5%) was recorded at fraction No. 4.

**Table 2:** Effect of different TLC extracted fractions of *B. bassiana* Si NPs on the percentage of larval mortality of *S. littoralis* after 24, 48 and 72 h

No. of fraction	Mean of % Larval mortality after 24, 48 and 72 h
**1**	16.42^c^
**2**	23.45^b^
**3**	39.84^a^
**4**	10.50^e^
**5**	13.37^d^
**Control**	0.00^f^
**F**	***
**LSD**	00.00

Values with different letters within the column are significantly different at *p* < 0.05; each value is the mean of 3 replicates

### Effects of AM, *S. littoralis, B. bassiana* Si NPs and Chlorpyrifos on morphological responses

**Fig. 2:**
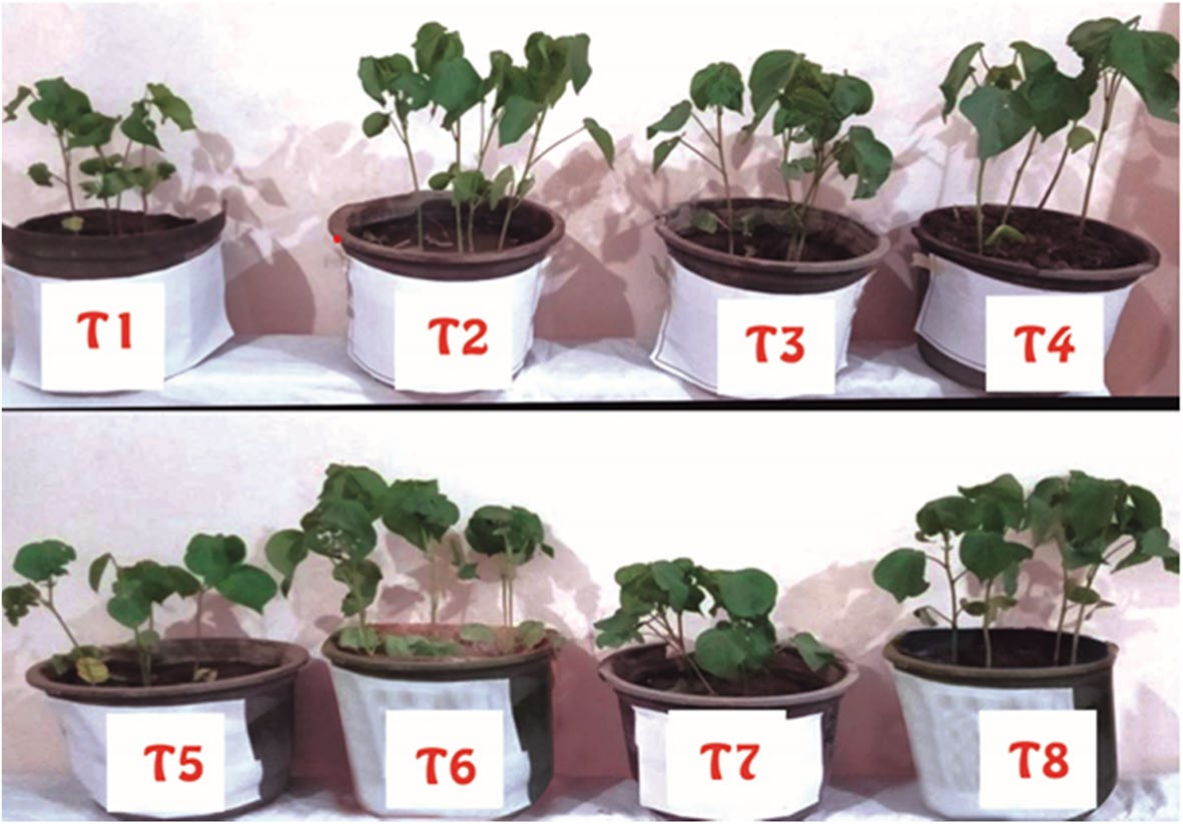
Photograph of the phenology of AM and non-AM cotton plants infested or not by *S. littoralis* and treated with Chlorpyrifos or *B. bassiana* Si NPs. T1 and T2 represent non-AM and AM control cotton plants without infestation; respectively. T3 and T4 represent non-AM and AM cotton plants infested with *S. littoralis;* respectively*.* T5 and T6 represent non-AM and AM cotton plants infested with *S. littoralis* and treated with *B. bassiana* Si NPs; respectively. T7 and T8 represent non-AM and AM cotton plants infested with *S. littoralis* and sprayed with Chlorpyrifos insecticide; respectively

**Fig. 3:**
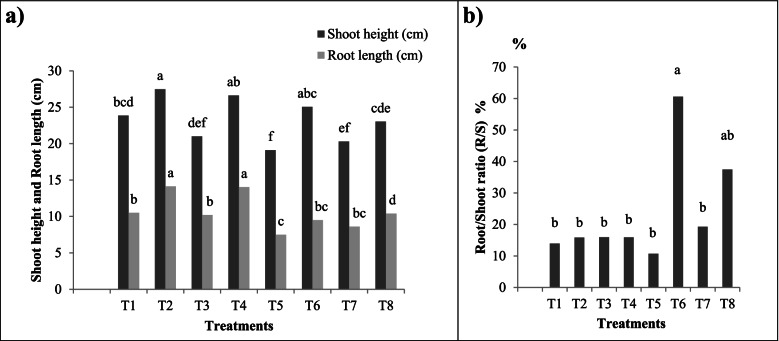
**a** Shoot height and root length; **b** Root/Shoot ratio (R/S) of AM and non-AM cotton plants infested or not by *S. littoralis* and treated with Chlorpyrifos or *B. bassiana* Si NPs. *T1 and T2 represent non-AM and AM control cotton plants without infestation; respectively. T3 and T4 represent non-AM and AM cotton plants infested with *S. littoralis;* respectively*.* T5 and T6 represent non-AM and AM cotton plants infested with *S. littoralis* and treated with *B. bassiana* Si NPs; respectively. T7 and T8 represent non-AM and AM cotton plants infested with *S. littoralis* and sprayed with Chlorpyrifos insecticide; respectively. *Values with different letters within the same column are significantly different at *p* < 0.05; each value is the mean of 5 replicates

Figure 2 showed the phenology of AM and non-AM cotton plants sprayed with Chlorpyrifos or *B. bassiana* Si NPs either in the presence or absence of *S. littoralis*. Infestation of cotton plants with *S. littoralis* had an inhibitory effect on growth parameters (Table 3 and Fig. 3). In AM plants, these growth parameters were considerably amplified compared to non-AM ones. Where, under control conditions (non-pest infestation), shoot Fwt of AM cotton plants were significantly (*p* < 0.05) improved (4.9 g/plant) compared to non-AM ones (3.6 g/plant). Moreover, our findings revealed that with AM inoculation an increase in all growth parameters of cotton plants treated with *B. bassiana* Si NPs as compared to non-AM ones.

**Table 3:** Fresh (Fwt) and dry weights (Dwt) of mycorrhizal (AM) and non-mycorrhizal shoots and roots of cotton plants reared with (+) or without (−) *S. littoralis* under different treatments

Treatments	Fwt (g/plant)			Dwt (g/plant)			MD %
	Shoot	Root	Total	Shoot	Root	Total	
**-** ***S. littoralis***	3.638^bc^	0.539^ab^	4.178^bc^	1.044^ab^	0.145^b^	1.189^ab^	–
***- S. littoralis +*** **AM**	4.906^a^	0.674^a^	5.581^a^	1.342^a^	0.204^ab^	1.546^a^	23.07^c^
***+S. littoralis***	3.020^c^	0.520^ab^	3.540^c^	0.710^bc^	0.113^b^	0.823^bc^	–
***+S. littoralis*** **+ AM**	4.501^ab^	0.591^ab^	5.092^ab^	0.950^abc^	0.140^b^	1.090^abc^	24.48^b^
***+ S. littoralis + B. bassiana*** **Si NPs**	2.953^c^	0.360^b^	3.313^c^	0.675^bc^	0.0720^b^	0.747^bc^	–
***+ S. littoralis + B. bassiana*** **Si NPs + AM**	4.43^ab^	0.413^b^	4.843^ab^	0.700^bc^	0.440^a^	1.140^abc^	34.50^a^
***+S. littoralis*** **+ Chlorpyrifos**	2.961^c^	0.450^ab^	3.411^c^	0.590^c^	0.104^b^	0.694^c^	–
***+S. littoralis*** **+ Chlorpyrifos + AM**	4.303^ab^	0.492^ab^	4.795^ab^	0.651^bc^	0.243^ab^	0.894^bc^	22.31^d^
**F**	***	NS	***	NS	**	**	***
**P**	0.000	0.2540	0.0000	0.0691	0.0027	0.0091	0.0000

Values with different letters within the same column are significantly different at *p* < 0.05; each value is the mean of 5 replicates. - *S. littoralis* and + *S. littoralis* refer to cotton plants non-infested or infested by *S. littoralis.* AM: cotton plants inoculated with AM fungi. *B. bassiana* Si NPs refers to cotton plants treated with *B. bassiana* Si NPs

Likewise, Table 3 showed that there was a slight decrease in morphological parameters of *S. littoralis* infested cotton plants with Chlorpyrifos compared to infested control*.* Remarkably, there was an increase in shoot Fwt and root Dwt of cotton reared with *S. littoralis* with the dual application of AM fungi and *B. bassiana* Si NPs or Chlorpyrifos insecticide (Table 3 and Fig. 3)*.* Besides, Fig. 3 showed that the highest reading of root/ shoot (R/S) ratio was recorded in AM cotton plants reared with *S. littoralis* and treated with *B. bassiana* Si NPs or Chlorpyrifos compared to other treatments.

### Influences of *S. littoralis, B. bassiana* Si NPs and Chlorpyrifos on mycorrhizal colonization and mycorrhizal dependency (MD)

**Fig. 4:**
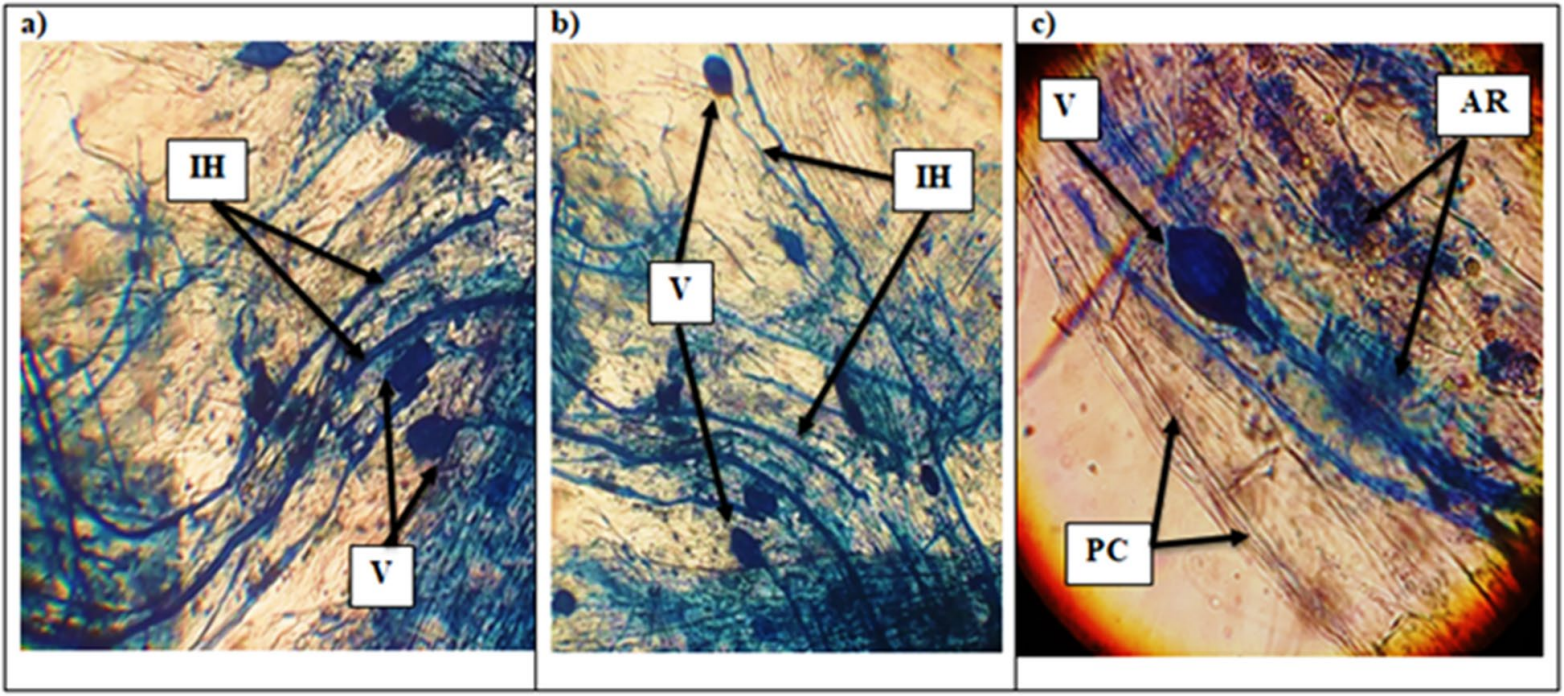
Photomicrographs of structural colonization of AM fungi in the roots of cotton plants: **a** and **b** Vesicles (V) and intraradical hypha (IH); **c** Vesicles (V), arbuscules (AR) and plant cell (PC) of cotton root

Table 4 represented the different levels of AM colonization in control and *S. littoralis* infested cotton roots treated with Chlorpyrifos or *B. bassiana* Si NPs. Distinctive mycorrhizal colonization structures [intercellular and intracellular hyphae (IH), vesicles (V), arbuscules (AR)] were observed in cotton roots (Fig. 4). Firstly, AM roots of non-infected control cotton plants showed high levels of the evaluated colonization parameters [colonization frequency (F%) and intensity (M%), and arbuscules frequency (A%)], recording 96.67, 47.07, and 26.37%, respectively. Also, a reduction in mycorrhizal colonization levels in cotton roots infected by *S. littoralis* compared to those non-infested. Whereas, no mycorrhizal colonization was detected in non-AM cotton plants.

**Table 4:** Frequency of mycorrhizal colonization (F%), intensity of mycorrhizal colonization (M%), arbuscular frequency (A%) of AM inoculated cotton plant roots reared with (+) or without (−) *S. littoralis* under different treatments

Treatments	Mycorrhizal colonization levels (%)		
	F	M	A
**-** ***S. littoralis***	0.0^b^	0.0^c^	0.0^d^
*-* ***S. littoralis +*** **AM**	96.67^a^	47.07^a^	26.37^a^
***+S. littoralis***	0.0^b^	0.0^c^	0.0^d^
***+S. littoralis*** **+ AM**	90.00^a^	40.83^ab^	21.40^b^
***+ S. littoralis + B. bassiana*** **Si NPs**	0.0^b^	0.0^c^	0.0^d^
***+ S. littoralis + B. bassiana*** **Si NPs + AM**	89.00^a^	37.21^b^	18.97^bc^
***+S. littoralis*** **+ Chlorpyrifos**	0.0^b^	0.0^c^	0.0^d^
***+S. littoralis*** **+ Chlorpyrifos + AM**	95.33^a^	34.37^b^	15.47^c^
**F**	***	***	***
**P**	0.000	0.000	0.000

Values with different letters within the same column are significantly different at *p* < 0.05; each value is the mean of 5 replicates. - *S. littoralis* and + *S. littoralis* refer to cotton plants non-infested or infested by *S. littoralis;* respectively*.* AM, cotton plants inoculated with AM fungi. *B. bassiana* Si NPs refers to cotton plants treated with *B. bassiana* Si NPs*.* F% represents frequency of root colonization, M% represents intensity of mycorrhizal colonization in root tissues and A% represents the level of arbuscular formation in root segments

Secondly, our results showed a decrease in colonization levels in *S. littoralis* infected cotton roots with Chlorpyrifos and *B. bassiana* Si NPs compared to AM control cotton roots. Moreover, our results in Table 4 revealed a close link between the intensity of cortical infection (M%) and total biomass of control or *S. littoralis* infested cotton plants under Chlorpyrifos and *B. bassiana* Si NPs applications. The noteworthy result was that with *B. bassiana* Si NPs application, MD of *S. littoralis* infested cotton plants increased significantly compared with control.

### Effects of AM fungi, *B. bassiana* Si NPs and Chlorpyrifos on total soluble carbohydrates and protein contents

Table 5 showed the effects of AM fungal inoculation on total soluble carbohydrates and protein contents of *B. bassiana* Si NPs or Chlorpyrifos treated cotton plants under leafworm, *S. littoralis* infestation. Generally, the infestation of cotton plants with *S. littoralis* resulted in a drastic decline in both carbohydrates and protein contents of AM and non-AM cotton plants. Though, AM cotton plants infested with *S. littoralis* exhibited an increase in sugar content as compared to that of non-AM infested ones. Likewise, it was apparent that the use of Chlorpyrifos caused a noticeable decrease in total soluble carbohydrates and protein of *S. littoralis* infested cotton leaves and the decrease was more apparent in non-AM colonized plants (25.45%) compared to control ones (Table 5). However, with AM fungal inoculation, 23.25, 15.5 and 11% of the increase in carbohydrates content were detected in non-infested or infested cotton plants and sprayed with *B. bassiana* Si NPs and Chlorpyrifos, respectively. Nevertheless in AM plants, protein contents, did not highly affect, remained around the control value in comparison with non-AM ones.

**Table 5:** Total soluble carbohydrates (mg/g Dwt), protein content (mg/g Fwt) and lipid peroxidation (nM/g Fwt) of mycorrhizal (AM) and non-mycorrhizal cotton plants grown under *S. littoralis* pest stress conditions

Treatments	Total soluble carbohydrates (mg/g Dwt)	Total soluble protein content (mg/g Fwt)	MDA content (nM/g Fwt)
**-** ***S. littoralis***	48.888^b^	2.584^a^	0.092^c^
***- S. littoralis +*** **AM**	60.255^a^	2.652^a^	0.059^c^
***+S. littoralis***	33.366^d^	1.342^b^	1.887^ab^
***+S. littoralis*** **+ AM**	35.199^c^	1.398^b^	1.017^bc^
***+ S. littoralis + B. bassiana*** **Si NPs**	26.827^g^	1.265^b^	2.578^a^
***+ S. littoralis + B. bassiana*** **Si NPs + AM**	30.983^e^	1.312^b^	2.313^ab^
***+S. littoralis*** **+ Chlorpyrifos**	24.872^h^	1.171^b^	2.683^a^
***+S. littoralis*** **+ Chlorpyrifos + AM**	27.622^f^	1.203^b^	2.526^a^
**F**	***	NS	NS
**P**	0.000	0.856	0.299

Values with different letters within the same column are significantly different at *p* < 0.05; each value is the mean of 5 replicates. - *S. littoralis* and + *S. littoralis* refer to cotton plants non-infested or infested with *S. littoralis;* respectively. AM, cotton plants inoculated with AM fungi. *B. bassiana* Si NPs refers to cotton plants treated with *B. bassiana* Si NPs

### Effects of AM fungi, *B. bassiana* Si NPs and Chlorpyrifos on lipid peroxidation (malondialdehyde content, MDA)

Regarding Table 5 results, *S. littoralis* infestation significantly induces an increase in MDA content of both AM and non-AM cotton plant leaves. Also, Chlorpyrifos and *B. bassiana* Si NPs resulted in a further increase in their content. However, AM fungal colonization reduced these contents in all treatments. The percent of the reduction in MDA content attributable to AM colonization reached 5.85 and 10.27% in plants treated with Chlorpyrifos and *B. bassiana* Si NPs, respectively.

### AM fungi, *B. bassiana* Si NPs and Chlorpyrifos effects on the non-enzymatic system (proline content)

**Fig. 5:**
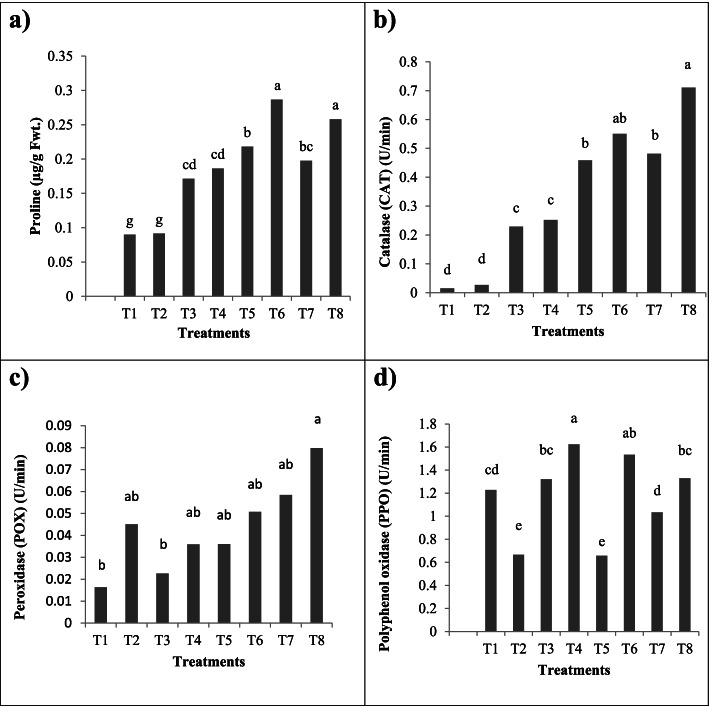
**a** Proline content (μg/g Fwt), (**b)** Catalase (CAT), **(c)** Peroxidase (POX) and **(d)** Polyphenol oxidase (PPO) enzymes activities of mycorrhizal (AM) and non-mycorrhizal (non-AM) cotton plant leaves infested with *S. littoralis* pest under different treatments. T1 and T2 represent non-AM and AM control cotton plants without infestation; respectively. T3 and T4 represent non-AM and AM cotton plants infested with *S. littoralis;* respectively*.* T5 and T6 represent non-AM and AM cotton plants infested with *S. littoralis* and treated with *B. bassiana* Si NPs; respectively. T7 and T8 represent non-AM and AM cotton plants infested with *S. littoralis* and sprayed with Chlorpyrifos insecticide; respectively.*Values with different letters within the same column are significantly different at *p* < 0.05; each value is the mean of 5 replicates

Figure 5 showed that the proline production was lower in non-infested control cotton plants and with *S. littoralis* infestation, an augmentation in its production was recorded. Also worth mentioning, AM fungal inoculation exhibited a marked increase in its content either in *S. littoralis* infested or non-infested cotton plants. Notable, with Chlorpyrifos or *B. bassiana* Si NPs, the effectivity of AM fungi was higher under infested conditions and produce higher proline as osmoregulant. Also, *S. littoralis* infested cotton plants and treated with Chlorpyrifos showed a significant enhancement in the proline content.

### AM fungi, *B. bassiana* Si NPs and Chlorpyrifos effects on detoxifying enzymes activity

Figure 5 showed that the antioxidant enzymes activity in AM and non-AM cotton plant leaves treated with Chlorpyrifos or *B. bassiana* Si NPs either in the presence or the absence of *S. littoralis* differed significantly from the control. Of note, the effectivity of AM fungi was high under infested conditions and induced significant antioxidant enzymes activity compared to non-AM ones under different treatments. It was noticeable that with *S. littoralis* infestation an increase in CAT, POX and PPO activities in cotton plant leaves was recorded reflecting the toxicity level and also the ability to combat the stress (Fig. 4).

Also, Fig. 5 demonstrated that AM fungi effectively enhanced CAT, POX and PPO activities in cotton plant leaves to scavenge the ROS and prevent the oxidative stress in plant cells. Furthermore, Chlorpyrifos application caused a significant enhancement in their activities in cotton plants. Noteworthy, with Chlorpyrifos or *B. bassiana* Si NPs, the effectivity of AM fungi was higher under infested conditions. Likewise, further augmentations in CAT, POX and PPO activities owing to the dual application of AM inoculation with Chlorpyrifos or *B. bassiana* Si NPs to *S. littoralis* infested cotton plants were recorded.

## Discussion

*B. bassiana* produces a diverse array of bioactive compounds known as secondary metabolic compounds; these metabolites have been shown to have potential insecticidal activity and might be used as biopesticides [37]. Ganesh and Murugan [38] showed that octadecenoic acid methyl ester and 2H-Pyran in *Sida cordata* extracts had antioxidant activities and thus protected plants. Also, Oleyl alcohol trifluoroacetate presented in ethyl acetate extract of *B. bassiana* Si NPs acts as nematicide and pesticide as reported by Sudha and Balasundaram [39] in *Padina pavonica* extract. As well, Mohana and Lavanya [40] highlighted that 2,2-Bis[4-[[4-chloro-6-(3-ethynylphenoxy)-1,3,5-triazin-2-yl]oxy] phenyl] propane had a low binding energy, that aids in the binding of the target molecule to pathogen receptors forming a strong ligand between them. Moreover, 2H-Pyran-tetrahydro-2-(12- pentadecynyloxy)- (CAS) and 9-octadecenoic acid-methyl ester-(CAS) had inhibitory effects on acetylcholine esterase (AChEI) and cyclooxygenase enzymes of the cell [41]. In addition, biological activities of octadecadienoic acid and 1,2-15,16- Diepoxyhexadecane enable them to be utilized in the development of novel formulations for pathogens and pests control and exhibited considerable effects on the rice pest, *Sitophilus oryzae*. Their insecticidal activities might be due to the shutdown of different biosynthetic routes of the pest’s metabolic pathways that can inhibit the feeding behaviour and growth regulators, disrupting the endocrinological balance of the insects [42].

There are no earlier reports on using organic extracts of *B. bassiana* Si NPs for *S. littoralis* control. Yet, several studies are showing the effect of only fungal organic extracts on insects. AL-Mekhlafi [43] reported that *Paecilomyces lilacinus* methanolic extract caused 100% mortality against *Aedes caspius* after 1 day. Likewise, spores of *Metarhizium anisopliae* resulted in 98% mortality in spotted spider mite, *Tetranychus urticae* after 1 week [44]. As well, Elbanhawy et al. [13] stated that the *Purpureocillium lilacinum* methanolic extracts from the spores and mycelia displayed insecticidal activity against *Aphis gossypii*. Also, Xu et al. [3] confirmed that *B. brongniartii*-Fe^0^ NPs caused significant reductions in feeding and growth parameters of *S. litura*. Besides, wang et al. [22] evinced that *Isaria fumosorosea*-Fe^0^ NPs application resulted in a 68% reduction in egg hatchability of sweet potato whitefly. Furthermore, our previous findings showed that applying *B. bassiana* metabolites pretreated with TiO_2_ NPs was the most effective on most biological aspects of *S. littoralis*, demonstrating insecticidal activity in larval, pupal, and adult stages of *S. littoralis* [45], and we concluded that treating *B. bassiana* with TiO_2_ NPs increased its activity allowing them to be used directly as a biological control agent against *S. littoralis*.

Growth parameters such as shoot and root Fwt and Dwt can be used to show the toxicity of *S. littoralis* infestation and chemical pollutants such as pesticides [46]. Infestation of cotton plants with *S. littoralis* had an inhibitory effect on growth parameters; this result was in accordance with Kousar et al. [47] in tomato plants infested with *S. litura*. Moreover, our findings revealed that with AM inoculation an increase in all growth parameters of cotton plants treated with *B. bassiana* Si NPs as compared to non-AM ones. Such enhancing effects of AM fungi were in line with Lin et al. [48] on *Leymus chinensis*, as AM fungi enhance the production of endogenous hormones that improve the metabolism [49]. Likewise, in the presence of AM fungi, cotton plants will have the adaptability to critical sites since they have improved tolerance to stress conditions and are able to resist the invading pathogens as well as acquire more nutrients through their hyphal network and thus increase their growth [1, 25, 50].

Furthermore, the slight decrease in morphological parameters of *S. littoralis* infested cotton plants with Chlorpyrifos corroborates with *Parween* et al. [33] *and* Fatma et al. [51] *with Chlorpyrifos and* mancozeb *exposure in Vigna radiata and Allium cepa plant.* Growth retardation owing to Chlorpyrifos indicates that this insecticide can probably harm the cotton by posing stress and overwhelmingly compromising plant cellular functions, reduction in cell division, cell elongation and conversion of indole-3 acetic acid into various photo-oxidative products [5, 52]. Remarkably, the increase in shoot Fwt and root Dwt of cotton reared with *S. littoralis* with the dual application of AM fungi and *B. bassiana* Si NPs or Chlorpyrifos insecticide may be a result of *B. bassiana* Si NPs or Chlorpyrifos insecticide effect in reducing the insect damage caused by *S. littoralis.* In addition, the effective role of AM fungal inoculation in strengthening the cotton plant’s health through facilitating the nutrient uptake from the soil [26, 53] and helping the plants better cope with *S. littoralis* pest stress through increasing its resistance and activation of plant immune responses [27, 34]. Furthermore, the highest R/S ratio was recorded in AM cotton plants reared with *S. littoralis* and treated with *B. bassiana* Si NPs or Chlorpyrifos, this indicated a significant phytotoxic nature of *S. littoralis* and Chlorpyrifos [51].

AM fungal inoculation is very important for cotton production as cotton is AM dependent crop [1, 54]. The decrease in AM colonization levels in *S. littoralis* infected cotton roots with Chlorpyrifos and *B. bassiana* Si NPs corroborated with Jin et al. [55] and Yadav and Aggarwal [56] that Metalaxyl and Benomyl had a marked inhibitory effect on AM fungal root colonization in pea, chickpea and *Helianthus annuus*. Contrary to our results, Metwally and Abdelhameed [5] proclaimed that mycorrhizal colonization was encouraged by Metalaxyl, Ridomil and Bavistin in cucumber roots. The discrepancies between our reports and those of the above studies are hard to appreciate, but may in part be attributable to differences in the AM fungal species concerned, which respond differently to pesticides. Other aspects such as the plant species involved, pesticide concentration applied and conditions of growth may also contribute to such variations [4].

Also, the reduction in colonization rates with *B. bassiana* Si NPs application may be a result of *S. littoralis* pathogenicity or antifungal activity of Si NPs against AM fungi. This was compatible with Jo et al. [57] results that the antifungal activity of Ag NPs against *B. sorokiniana* and *M. grisea* could be attributed to the direct contact of Ag with the spores thus affecting the formation of germ tubes as well as conidia germination. Also, Nair et al. [58] stated that the toxic effect of NPs may be due to the ability of NPs to accumulate and penetrate more than normal molecules causing membrane dysfunction. Conversely, Farias et al. [59] stated that F% in AM soybean and sugarcane inoculated with *B. bassiana, Metarhizium anisopliae* and *Purpureocillium lilacinum* was 20% higher for soybean and 28% higher for sugarcane in comparison to the non-inoculated ones. However, in another study, it was revealed that there is no significant difference in soil fungal community composition of tomato rhizosphere associated with the application of *Paecilomyces lilacinus* [60].

Also, with *B. bassiana* Si NPs application, MD an indicator of how a plant is dependent on AM fungi to produce its maximum growth [61] of *S. littoralis* infested cotton plants increased significantly compared with control. These findings agree with Metwally and Abdelhameed [34] and Rabie [62], where under stress; enhanced growth of cotton plants extremely depends on AM colonization and thus increased the MD values. Thus AM helped cotton plants to overawed the detrimental effect of pest and pesticides stress indicating of the ecological significance of AM colonization for plant survival and growth under stress [34, 63].

Sugars are the primary photosynthetic products that form the building blocks for all other chemical components of plants [64], as well protein is a significant parameter for the determination of phytotoxicity caused by environmental stresses [32]. Soluble carbohydrates and proteins in plants under a pathogen attack may be variously modified, both by plant regulatory mechanisms and by pathogen interference. The drastic decline in carbohydrates and protein contents with *S. littoralis* infestation was observed, although AM colonization increased sugar content compared to non-AM infested ones. This result was coherent with Kousar et al. [47] that infestation of tomato plants with *S. litura* caused a significant decrease in total protein and sugars, but with plant growth promoting rhizobacteria (PGPR) application a significant increase was recorded. There are several causes for qualitative changes of sugars and proteins at the infection site, Kousar et al. [47] and Chen et al. [65] reported that the level of sugars is reduced through their consumption for energy and structural purposes or through their uptake by the pathogen, while it happens in autotrophic tissues due to inhibition of photosynthesis. Also, the decrease in protein content could be explained that when the plant cell is under stress, the signaling pathway sends signals to the nucleus of the cell. Due to this signaling, genes expression undergoes changes leading to changes in the amount and type of special proteins [66].

The decrease of soluble carbohydrates and protein with Chlorpyrifos coordinated with what Shakir et al. [32] and Huang and Xiong [67] stated in tomato seedlings and *Oryza sativa* treated with insecticides, Acetochlor and Bensulfuron methyl. This reduction may be a result of an osmotic shock of pesticides resulting in a release of protein and loss of membrane transportability in the leaf cells. Also, Chlorpyrifos reduced the ratios of NAD and NADP, the electron transport system interfered and the ATP levels decreased, leading to the diminution in the carbohydrates content. Our findings conflict with what Metwally and Abdelhameed [5] stated in *Cucumis sativus* plants treated with Agrothoate. However, with AM fungal inoculation, an increase in carbohydrates content was detected, this result could be attributed to enhancing photosynthesis and hydrolysis of starch to soluble sugar by AM and the sink effect of AM demanding sugars [5, 68].

Lipids play an important role in maintaining the structural integrity of cells as a fundamental component of cellular membranes, and lipid peroxidation reveals the severity of oxidative stress [64]. Malondialdehyde (MDA) is a measure of the increase in oxidative stress caused by various stressors such as herbivorous insects, which can harm plants by raising levels of ROS, which have negative effects on cell metabolism and biochemical processes. These ROS cause peroxidation of the membrane’s unsaturated lipid components, resulting in membrane integrity loss and, as a result, leakage and desiccation [69, 70].

*S. littoralis* infestation and Chlorpyrifos and *B. bassiana* Si NPs applications significantly increase the MDA content of both AM and non-AM cotton plant leaves; this pesticide caused oxidative stress in cotton leaves by ROS production that caused peroxidation in membranous lipids and the formation of MDA [5, 71]. However, AM fungal colonization reduces these contents in all treatments. This may be due to the substantial increase in antioxidant activities in AM plants leading to less lipid peroxidation where antioxidants scavenge the radical production before reacting with the membrane lipids and minimizing its peroxidation [72]. This result was well-matched with the results of Parween et al. [33] in *Vigna radiata* treated with Chlorpyrifos. Also, Abd-ur-Rahman et al. [73] exhibited a significant change in MDA concentration in *Brassica oleracea* infested with *S. litura* after application of Bifenthrin, Emamectin Benzoate and Lufenuron insecticides.

The accumulation of cellular osmolytes in plants helps in sustaining cellular function and physiological stability under stress including pathogen attacks [34, 74, 75]. One of these osmolytes is proline, a vast array of data suggests a positive correlation between its accumulation and plant stress [34, 47]. The augmentation in proline content with *S. littoralis* infestation may be due to the enrichment in proline synthesizing enzymes and reduction in catabolizing ones or its circumscribed assimilation in protein synthesis, a further increase was recorded with AM fungal inoculation. Kaur et al. [74] displayed a 1.2-fold increase in proline in wheat leaves infested by aphids as compared to the non-infested. Notable, with Chlorpyrifos or *B. bassiana* Si NPs, the effectivity of AM fungi was higher under infested conditions and produce higher proline as osmoregulant. Ullah et al. [75] indicated that the application of PGPR to maize plants displayed a substantial increase in proline content as compared to untreated plants. Moreover, Kousar et al. [47] affirmed that infestation of tomato plants with *S. litura* caused a significant increase in proline content in tomato leaves, and further augmentation in its content was recorded with the application of PGPR under both control and infested conditions.

Additionally, *S. littoralis* infested cotton plants and treated with Chlorpyrifos showed a significant enhancement in the proline content, similar records were observed by Parween et al. [33] on *Vigna radiata* in response to chlorpyrifos and by Wu et al. [76] on rice in response to insecticides. Also, Abd-ur-Rahman et al. [73] noticed an increase in proline content in *Brassica oleracea* infested with *S. litura* after application of Bifenthrin, Emamectin Benzoate and Lufenuron insecticides. These pesticides seem to have imposed oxidative stress on treated cotton plants, which is a rapid and sensitive response of plants to environmental stress.

Defense responses are often induced in the plants following the attack by pathogens or herbivore feeding. Once attacked by pests, plants initiate sophisticated defense responses including the regulation of defense pathways and production of defensive compounds which result in local or systemic resistance [6, 77]. Among these defensive compounds are antioxidant enzymes such as CAT, POX, and PPO. These enzymes play an important role in ROS scavenging and preventing their damaging effects on many sensitive molecules i.e. lipids, proteins and nucleic acids.

The increase in CAT, POX and PPO activities in cotton plant leaves with *S. littoralis* infestation was inconsistent with Mohamed et al. [6] who studied the anti-oxidative activities of soybean genotypes infested by *S. littoralis* and reported well-expressed CAT, POX and PPO in their shoots. Similarly, our findings are coherent with the previous studies of Kaur et al. [74] and Zhao et al. [77] that displayed a significant increase in CAT, POX and PPO activities in aphid and whitefly infested wheat and tobacco plants; respectively as compared to the respective non-infested ones. The increase in the activity of these enzymes in cotton plants owing to *S. littoralis* infection is a clear indication of the possible involvement of these enzymes in the plant detoxification mechanisms besides this anti-oxidative defense is a crucial part of the basic metabolism, empowering the plant to cope instantaneously with rapid environmental stresses.

POX plays a role in lignification and cell wall rigidity and protects the plants against infestation by herbivores [78]. Besides, POX may generate phenoxy and other oxidant radicals that can directly prevent the feeding of insect herbivores and produce toxins that decrease plant digestibility [79]. Also, Helmi and Mohamed [80] and Mohamed et al. [6] reported that CAT acts as anti-nutritional and/or toxicological defenses against insect herbivores. Moreover, PPO generates quinones which produce oxidative stress in the gut lumen of insects; these produced quinones along with ROS may be absorbed and have toxic effects on herbivores [80].

Figure 5 demonstrated that AM fungi effectively enhanced CAT, POX and PPO activities in cotton plant leaves to scavenge the ROS and prevent the oxidative stress in plant cells. Furthermore, Chlorpyrifos application caused a significant enhancement in their activities in cotton plants. A similar result was observed by Fatma et al. [51] in *Allium cepa* plants. These pesticides seem to have imposed oxidative stress on treated plants, which is a rapid and sensitive response of plants to environmental stress. There is some evidence of pesticide degradation by elevated activity of oxidoreductase enzymes [5, 51]. Noteworthy, with Chlorpyrifos or *B. bassiana* Si NPs, the effectivity of AM fungi was higher under infested conditions.

Also, the further augmentation in CAT, POX and PPO activities owing to the dual application of AM inoculation with Chlorpyrifos or *B. bassiana* Si NPs to *S. littoralis* infested cotton plants can lead to a higher ROS elimination that can denature different plant bio-molecules. Our results corroborate with Xu et al. [3] who reported that *B. brongniartii* Fe^0^ NPs enhanced the activities of CAT and POX. Similarly, a study by Sharma and Mathur [81] confirmed that PGPR alone and/or in association with fungi significantly enhanced the antioxidant enzymes in *Brassica juncea* infested with *S. litura* that lead to an enhanced immune system against herbivory. Also, Abd-ur-Rahman et al. [73] proclaimed an increase in POX activity in *Brassica oleracea* infested with *S. litura* after Bifenthrin, Emamectin Benzoate and Lufenuron insecticides application. As a result, the observed anti-oxidative enhancement in AM colonized cotton plants infested with *S. littoralis* is a mechanism to combat insect induced oxidative stress and help plants to maintain the ROS levels below their deleterious levels and mediate quick removal of toxic ROS so that metabolism remains stable [5].

## Conclusions

This study described the insecticidal bio-efficacy of AM fungi and *B. bassiana* Si NPs against *S. littoralis* infested cotton plants as a biological alternative solution to reduce the negative environmental consequences of chemical pesticides such as Chlorpyrifos. Our results showed that Oleyl alcohol, trifluoroacetate was the major compounds in ethyl acetate extract detected by Gas chromatography-mass spectrometry (GC–MS) analysis that acts as nematicide and pesticide. Also, the results exhibited the stimulatory effects of AM fungal inoculation on growth, biochemical parameters as well as the detoxifying mechanism in cotton plants treated with *B. bassiana* Si NPs or Chlorpyrifos under *S. littoralis* stress. From the above findings, it is suggested that AM fungi can potentially be used in environmentally friendly *S. littoralis* management. However, further work is required to determine the efficacy and persistence of AM fungi and *B. bassiana* Si NPs under field conditions.

## Methods

### Biological materials

#### *Spodoptera littoralis* (Boisd.) insect culture

The present study was carried out on the Egyptian cotton leafworm, *S. littoralis* (Boisd.) (Lepidoptera: Noctuidae). The original culture was obtained from a well-established culture reared at the Department of Cotton Leafworm, Plant Protection Research Institute, Sharkia Branch, Egypt. The maintained insect culture was reared under laboratory conditions of 26 ± 1 °C, 70 ± 5% humidity and 12 h/12 h of day/night cycle [82]. The newly hatched larvae from egg masses were maintained in glass jars and provided daily with castor bean leaves (*Ricinus communis*). Upon pupation, pupae were carefully collected. When adults emerged, they were sexed and kept in mating cages. Cotton wools soaked in 10% sugar solution were placed as wickers to provide sources of nutrition for moths and changed daily to avoid fermentation.

#### *Beauvaria bassiana* (Bals.) fungal metabolites based Si NPs

##### Preparation of *B. bassiana* based Si NPs

The fungal isolate of *B. bassiana* was obtained from Assuit University, Mycology Center, Egypt and was cultured on potato dextrose agar (PDA) medium for 2 weeks at 28 ± 1 °C. Spores were harvested and the spore suspension was cleaned from hyphal debris [83] and its concentration was adjusted to 2.7 × 10^7^ conidia/ mL [84]. The metabolites of *B. bassiana* applied with Si NPs were prepared by inoculating 1 mL of *B. bassiana* spore suspension and 1 mL of Si NPs solution (500 ppm) in an Erlenmeyer flask (250 mL) containing 100 mL sterile liquid Dox broth medium, then incubated at 28 ± 1 °C and 50–60% humidity for 7 days. Cell filtrate and mycelial mat were separated by using Whatman filter paper No.1 under the biosafety cabinet; therefore, the cell-free supernatant (CFS) became ready for further use and for foliar application.

#### AM fungal inoculum

Spores of AM fungi were isolated from rhizospheric soil from Sharkia Governorate, Egypt via wet sieving and decanting technique [85]. Approximately 200 g of air-dried soil was distributed in 2 L of water in a large jar and the suspension was left intact for 10–15 min. The suspension was then decanted 2–3 times through the stack of sieves of 400, 250, 180 and 38 μm in diameter. The residue from each sieve was collected into a small flask [86] and the morphology of AM fungal spores and sporocarps were observed and identified by using Manual for identification [87]. The mixture of identified spores of *Funneliformis mosseae, Funneliformis constrictum, Gigaspora margarita* and *Rhizophagus irregularis* together in pots filled with sterilized sandy clay soil were propagated on Sudan grass (*Sorghum sudanenses* Pers.) roots as an appropriate trap plant for inoculum production. AM inoculum consisted of AM spores, hyphae and colonized root fragments.

### Chemical pesticide (Chlorpyrifos [Dursban (48%EC)]

It is an organophosphorus insecticide supplied by Dow Agro Sciences with a chemical name of O, O-diethyl O-(3, 5, 6 -trichloro-2-pyridinyl) phosphorothioate (IUPAC). It inhibits the acetylcholine esterase (AChE) enzyme. It is used at the recommended dose of 5 mL /L of water.

### Experimental design (plant growth and Mycorrhizal inoculation)

A 2-year pot experiment was conducted in an environmental growth chamber under controlled conditions (16 h/8 h of day/night cycle, 30 ± 4 °C and 70–80% humidity) in the Botany and Microbiology greenhouse Department, Faculty of Science, Zagazig University, Egypt. Seeds of a variety Giza 86 of cotton (*Gossypium hirsutum* L. Merr.) obtained from Agriculture Research Center, Giza, Egypt, were surface sterilized with 7% sodium hypochlorite (NaOCl) for 10 min, subsequently rinsed with sterilized water and sown in sterilized plastic pots (28 cm diam. Top, 24 cm diam. Base and 22.5 cm depth) filled with 10 kg sterilized soil. The physicochemical characteristics of the soil were analyzed (Table 6) in the Central Laboratory of the Faculty of Agriculture, Zagazig University, Egypt. Two different treatments were administered to the potted plants as follows:

**Table 6:** Physicochemical attributes and nutritional status of the experimental soil before planting

Property	Sand	Silt	Clay	Soil texture	Anions (meq/1000 g soil)				Cations content (meq/1000 g soil)			Micronutrients (mg/ kg soil)				Organic matter (%)	Total P (%)	pH
	%				CO_3_^2−^	HCO^−^_3_	SO_4_^−^	Cl^−^	K^+^	Mg^2+^	Ca^2+^	Cu	Mn	Zn	Fe			
Value	9.7	27.5	62.8	Clay	ND	1.44	31.81	26.69	5.01	8.87	27.88	0.197	0.08	0.193	0.715	1.93	0.69	7.68

*ND Not Detected

1- Non-AM treatments: cotton seeds were planted in pots that received 100 g of sterilized soil per pot.

2- AM treatments: cotton seeds were planted in pots and received 100 g Sudan grass-root fragments per pot at the sowing date (approx. 80 spores/g trap soil).

Fifty days post sowing, plants were artificially infested using one egg mass of the cotton leafworm/ plant. When the 1st instar larvae emerged from egg masses on leaves, they were allowed to feed on plants till reached the 2nd instar larvae then the tested compounds (*B. bassiana* Si NPs and Chlorpyrifos) were sprayed. Each treatment was divided into four groups according to infestation with *S. littoralis* as follows:

**a-** The 1st group was left without infestation (control).

**b-** The 2nd group was artificially infested with *S. littoralis* and not sprayed either with Chlorpyrifos compound or *B. bassiana* Si NPs.

**c-** The 3rd group was artificially infested with *S. littoralis* and then sprayed with Chlorpyrifos compound.

**d-** The 4th group was artificially infested with *S. littoralis* and then sprayed with *B. bassiana* Si NPs.

There were five replicates (*n* = 5) for each treatment. The pots were arranged in a completely randomized design (2 × 4). The plants were harvested after 1-week spraying of the tested compounds to evaluate the morphological criteria and mycorrhizal status, and to conduct a variety of biochemical analyses.

### Measurements

#### Gas chromatography–mass spectrometry (GC–MS) analysis of the bioactive compounds from *B. bassiana* fungal extract

The bioactive metabolites of *B. bassiana* applied with Si NPs were extracted from CFS by liquid-liquid extraction as described by Chen et al. [88] using ethyl acetate 3 times via a separating funnel in a ratio of (0.5:1 (v∕v) solvent: filtrate) and sonicated for 15 min. The separated organic layer was collected and filtered over anhydrous sodium sulphate (Na_2_SO_4_). The extract was dried by a rotary evaporator (60–65 °C) to get a crude extract which was then separated by thin-layer chromatography (TLC) (Aluminum sheets 20 × 20 Si Gel 60 F_254_ Merck, Germany 0.25 mm) [89] using hexane/ ethyl acetate (1:9 v/v) solvent and the separated fractions were visualized using UV-Visible Spectrophotometer, RIGOL (Model Ultra-3660) at 254 and 366 nm. The most bioactive fraction was detected according to its larval mortality efficiency against the 2nd instar larvae of *S. littoralis* using leaf-dip bioassay [90] using treated castor bean leaves, *Ricinus communis*. The percentages of larval mortality were calculated after 24, 48 and 72 h post-treatment to determine the most bioactive fraction that was afterwards analyzed by GC–MS at the National Research Center, Cairo, Egypt using G/C/MS-QP − 1000– Ex gas chromatograph-Mass spectrophotometer (SHIMADZU-Japan) (Fig. 1) and comparing the unknown component with the spectrum of the known one stored in Chemical Abstracts Service (CAS) library. The name, molecular weight and molecular formula of the test material were ascertained (Table 1).

#### Morphological assessments

The whole cotton plants were removed from the soil, their shoots were detached and their roots were washed separately to remove soil particles and their fresh weights (Fwt) were measured. Subsequently, after drying in an electric oven at 80 °C for 48 h, the shoot and root dry weights (Dwt) were registered. Fwt and Dwt were measured as the average in gram per plant (g/plant). Also, total Fwt and Dwt of cotton shoots and roots as well root/ shoot ratio (R/S) were calculated.

#### AM fungal colonization percentages and mycorrhizal dependency (MD)

After 1 week of spraying of *B. bassiana* Si NPs and Chlorpyrifos, the roots of AM colonized cotton plants from different treatments were washed separately with tap water then cut into small pieces of 0.5–1 cm in length, then cleared with 10% KOH. The colonization percentage was evaluated by staining with 0.05% trypan blue for 15 min at 90 °C [91, 92]. For the evaluation of AM colonization, 40 segments of stained cotton roots were placed vertically on microscope slides with a few drops of lactoglycerol to facilitate the observation of the internal tissues. Afterwards, the mounted roots were detected with an optical microscope at 10X and AM colonization levels were assessed [93] according to frequency (F%) and colonization intensity (M%) as well as the level of arbuscular development (A%) by using the Mycocalc software. The photograph was taken to show hyphae, arbuscules and vesicles of AM fungi. Moreover, MD of *Gossypium hirsutum* was calculated using the following equation:

$$\mathrm{MD}=1-\frac{\left(\mathrm{mean}\ \mathrm{total}\ \mathrm{biomass}\ \mathrm{of}\ \mathrm{plants}\ \mathrm{with}\mathrm{out}\ \mathrm{AM}\ \right)}{\left(\mathrm{mean}\ \mathrm{total}\ \mathrm{biomass}\ \mathrm{of}\ \mathrm{plants}\ \mathrm{inoculated}\ \mathrm{with}\ \mathrm{AM}\right)}\kern0.5em \times 100$$

### Analyses of biochemical parameters of cotton plants

#### Total soluble carbohydrates and protein content

Carbohydrates content was estimated in 0.1 g Dwt of cotton leaves by phenol sulphuric acid method [94] at 490 nm after extracting with 2.5 N HCl in a boiling water bath for 3 h. The carbohydrates content was calculated in terms of μg/mL using the glucose standard curve and then expressed as mg/g Dwt.

$$\mathrm{mg}/\mathrm{g}\ \mathrm{Dwt}=\frac{\upmu \mathrm{g}/\mathrm{mL}\ \mathrm{x}\ \mathrm{Extract}\ \mathrm{volume}\ \mathrm{x}\ \mathrm{Dilution}\ \mathrm{factor}}{\mathrm{Dry}\ \mathrm{Weight}\ \left(\mathrm{Dwt}\right)\mathrm{of}\ \mathrm{sample}\ \left(\mathrm{g}\right)\mathrm{x}\ 1000}$$

Total protein content was estimated in 0.5 g Fwt of cotton plant leaves after homogenizing in 10 mL of 25 mM Tris – HCl buffer solution (pH, 8.5) [95] and its absorbance was measured at 595 nm. The protein concentrations were expressed as μg/g Fwt using Bovine serum albumin as standard.

#### Determination of lipid peroxidation

Lipid peroxidation was determined by measuring malondialdehyde (MDA) formation by the thiobarbituric acid method designated by Ohkawa et al. [96] after homogenizing 0.2 g Fwt of cotton leaf in 4 mL of 5% trichloroacetic acid (TCA) solution. The absorbance was recorded at 530 nm and corrected for nonspecific turbidity by subtracting the absorbance at 600 nm.

#### Proline content assessment

Proline content in a known Fwt of AM and non-AM cotton plant leaves was determined after homogenizing in 10 mL of 3% aqueous sulphosalicylic acid [97]. Equal volumes of glacial acetic acid, acidic ninhydrin and the filtrate were heated for 1 h in a boiling bath of water. The reaction was stopped by placing it in an ice bath, and then 4 mL toluene was added to the reaction mixture. The absorbance of the toluene layer was measured at 520 nm. The amount of proline was calculated from the standard curve of proline and then expressed on Fwt basis as follows:

$$\upmu \mathrm{moles}/\mathrm{g}\ \mathrm{Fwt}=\frac{\left(\upmu \mathrm{g}\ \mathrm{proline}\times \mathrm{mL}\ \mathrm{toluene}\times 5\right)}{\left(115.5\times \mathrm{g}\ \mathrm{sample}\ \mathrm{Fwt}\right)}$$

*Where 115.5 is the molecular weight of proline.

#### Assay of the activities of the defense-related enzymes: (catalase, peroxidase and polyphenol oxidase)

A known Fwt of cotton leaves (0.5 g) was homogenized in 4 mL of ice-cold 50 mM potassium phosphate buffer (pH, 7.0) containing 1 mM EDTA. The homogenate was centrifuged at 10000 rpm at 4 °C for 10 min and the supernatant was used for determining the activities of catalase (CAT) at 240 nm [98], peroxidase (POX) at 470 nm over 2 min interval [99] and polyphenol oxidase (PPO) [100]. Specific activity was defined as the unit of enzyme activity per min (U/ min).

### Statistical analysis

Each result was presented as the mean of 5 replicates. The significant differences between treatments were statistically evaluated by Tukey,s HSD test and two-way analysis of completely randomized (ANOVA) using Costat statistical software (2005) program. *p* < 0.05 was considered to be significant.

## Data Availability

All data generated or analyzed during this study are included in this published article.

## Abbreviations

AM: Arbuscular mycorrhizae
CAT: Catalase
CFS: Cell-free supernatant
Dwt: Dry weight
Fwt: Fresh weight
MD: Mycorrhizal dependency
Si NPs: Silica nanoparticles
PGPR: Plant growth promoting rhizobacteria
POX: Peroxidase
PPO: Polyphenol Oxidase
ROS: Reactive oxygen species
